# Gut microbiota and voluntary alcohol consumption

**DOI:** 10.1038/s41398-022-01920-2

**Published:** 2022-04-07

**Authors:** L. Segovia-Rodríguez, V. Echeverry-Alzate, I. Rincón-Pérez, J. Calleja-Conde, K. M. Bühler, E. Giné, J. Albert, J. A. Hinojosa, E. Huertas, F. Gómez-Gallego, C. Bressa, F. Rodríguez de Fonseca, J. A. López-Moreno

**Affiliations:** 1 Department of Psychobiology and Methodology in Behavioral Sciences, Faculty of Psychology, Somosaguas Campus, Complutense University of Madrid, 28223 Madrid, Spain; 2 IMABIS Foundation, Regenerative Medicine Laboratory, Carlos Haya Regional University Hospital, 29010 Málaga, Spain; 3 School of Life and Nature Sciences, Nebrija University, 28248 Madrid, Spain; 4 Multidisciplinary Institute, Complutense University of Madrid, 28040 Madrid, Spain; 5 Department of Cell Biology, Faculty of Medicine, Complutense University of Madrid, 28040 Madrid, Spain; 6 Department of health and Biological Psychology, Faculty of Psychology, Autonomous University of Madrid, 28049 Madrid, Spain; 7 Faculty of Psychology, Somosaguas Campus, Complutense University of Madrid, 28223 Madrid, Spain; 8 Faculty of Languages and Education, University of Nebrija, 28015 Madrid, Spain; 9 Department of Experimental Psychology, Cognitive Processes and Speech Therapy, Faculty of Psychology, Somosaguas Campus, Complutense University of Madrid, 28223 Madrid, Spain; 10 Faculty of health science, International University of La Rioja, 26006 Logroño, Spain; 11 Faculty of Experimental Sciences, Universidad Francisco de Vitoria, 28223 Madrid, Spain

**Keywords:** Addiction, Physiology

## Abstract

Alcohol is part of the usual diet of millions of individuals worldwide. However, not all individuals who drink alcohol experience the same effects, nor will everyone develop an alcohol use disorder. Here we propose that the intestinal microbiota (IMB) helps explain the different consumption patterns of alcohol among individuals. 507 humans participated in this study and alcohol consumption and IMB composition were analyzed. On the other hand, in 80 adult male Wistar rats, behavioral tests, alcohol intoxication, fecal transplantation, administration of antibiotics and collection of fecal samples were performed. For identification and relative quantification of bacterial taxa was used the bacterial 16 S ribosomal RNA gene. In humans, we found that heavy episodic drinking is associated with a specific stool type phenotype (type 1, according to Bristol Stool Scale; *p* < 0.05) and with an increase in the abundance of *Actinobacteria* (*p* < 0.05). Next, using rats, we demonstrate that the transfer of IMB from alcohol-intoxicated animals causes an increase in voluntary alcohol consumption in transplant-recipient animals (*p* < 0.001). The relative quantification data indicate that the genus *Porphyromonas* could be associated with the effect on voluntary alcohol consumption. We also show that gut microbiota depletion by antibiotics administration causes a reduction in alcohol consumption (*p* < 0.001) and altered the relative abundance of relevant phyla such as *Firmicutes*, *Bacteroidetes* or *Cyanobacteria* (*p* < 0.05), among others. Benjamini–Hochberg false discovery rate (FDR) correction was performed for multiple comparisons. These studies reveal some of the consequences of alcohol on the IMB and provide evidence that manipulation of IMB may alter voluntary alcohol consumption.

## Introduction

Intestinal microbiota (IMB) is the set of microorganisms (bacteria, fungi, yeasts and viruses, among others) that inhabit our intestines [1]. In most cases, when referring to IMB, one usually refers to the populations of bacteria that have colonized our large intestine. It has been estimated that the number of bacteria in this area of the intestine exceeds many thousand times the number of bacteria found in the small intestine [2, 3]. These bacteria have the potential to modulate the “biochemical language” used by our nervous system. For instance, gut bacteria can release neurotransmitters, their precursors or can even stimulate the release of brain neurotransmitters, and cause the release of hormones, cytokines and bacterial metabolites into the bloodstream [4–7]. The relevance of IMB lies in the host’s health. Although it has been shown that the absence of gut bacteria is compatible with life [8], many studies have demonstrated the role of IMB in several diseases, including mental health disorders (for a review see refs. [9, 10]). This indicates that variations in the composition of IMB can determine the host’s health and how the body adapts and responds to its environment.

Within this variability of how we adapt to the environment is the behavior of alcohol consumption. Alcohol is a regular part of the diet of millions of individuals around the world, and along with caffeine, alcohol is the most accessible drug in all age ranges [11]. However, some individuals seem to like more alcohol, get fewer negative effects after consumption or have a greater tolerance compared to other individuals who tend to avoid its consumption [12–14]. Multiple factors may explain the motivation for alcohol consumption or the development of alcohol use disorders, including environmental and biological factors [15–17]. Concerning this, in recent years the role of the IMB on alcohol consumption has been studied [18], showing an association through the immune system [19]. Therefore, this study aimed to contribute to the study of this relationship by results obtained in humans and rats, using bacterial next generation sequencing to explore the association between IMB and alcohol consumption.

## Materials and methods

### Human participants and sample collection

Alcohol consumption was assessed in 507 university students (83.3% women, mean age 19.8 ± 1.9) through a questionnaire asking the type of alcoholic beverage, the amount consumed, and the time spent in each episode of drinking. With this information and the weight of the participants, it was possible to estimate the grams of alcohol per kilogram of body weight consumed per week. The questionnaire also asked about other variables that could determine the exclusion of a participant for the study of IMB, for example, the use of antibiotics, drugs of abuse (except tobacco), drugs, pre/probiotics, metabolic disorders such as obesity (body mass index >30 kg/m^2^) and diabetes, inflammatory bowel disease, etc. Then passed the Bristol Stool Scale and a general questionnaire of food consumption. Human fecal samples were collected in a sterile container and immediately stored at −20 °C for less than 24 hours, and then stored at −80 °C until further processing [20, 21]. All procedures were in accordance with the Declaration of Helsinki and approved by the local ethical committee (CEI-72-1300; Autonomous University of Madrid) and all subjects signed an informed consent form.

### Animal experiments

Eighty adult male Wistar rats (Envigo, Barcelona, Spain) were used. The animals were separated into two general groups: donors (*n* = 14) and recipients (*n* = 66). The animals weighed 340–450 g at the start of the procedures. Animals were individually housed in a temperature and humidity-controlled environment (21 ± 1 °C), on a 12-h reverse light/dark cycle (lights off at 08:00 AM). Experimental sessions were performed during the dark phase. The animals were habituated to our facilities for 10 days before any procedure. All research was conducted in strict adherence to the European Directive 2010/63/EU and Royal Decree 53/2013 on the protection of animals used for scientific purposes. The Ethics Committee of the Faculty of Psychology of the Complutense University of Madrid and Autonomous Community of Madrid (PROEX 262/19) approved the study.

### Alcohol intoxication

Eight donor animals were subjected to alcohol dependence induction via intragastric alcohol administration and six donor rats served as controls. Alcohol solution was administered four times per day, at four h intervals (for a total of 10 g/kg/day), for 10 consecutive days. Alcohol solution was prepared by diluting 96% alcohol in a solution consisting of powdered milk (baby formula, Nativa 2, Nestlé^®^), sucrose, and water. This solution is intended to reduce gastrointestinal irritation caused by repeated alcohol intoxications [22]. Six donor rats served as controls and received intragastric administration of the solution of milk, sucrose and water. The withdrawal signs were examined before the first administration in both groups during the first six days. We used a withdrawal rating scale that included ventro-medial limb retraction (VLR), irritability to touch (vocalization), tail rigidity, and body tremors. Each sign was assessed from 0 to 2 (0 = no sign, 1 = moderate, 2 = severe) [23].

### Antibiotic cocktail administration

Neomycin (250 mg kg^−1^) (Sigma-Aldrich, Madrid, Spain), metronidazole (50 mg kg^−1^) (Sanofi, Gentilly, France) and polymyxin B (9 mg kg^−1^) (MedChemTronica, Stockholm, Sweden) were dissolved in sterile water and administered orally in a volume of 4 ml kg^−1^ [24]. The antibiotic cocktail (ABX) was prepared every day of treatment. To facilitate bacterial colonization through fecal transplantation, ABX was administered to 33 recipient animals (ABX group) for five consecutive days before the start of fecal transplants. The group of recipient animals that were not treated with ABX (No ABX group, *n* = 33) received sterile water.

### Fecal transplant

Fecal samples from days 5 to 10 of alcohol intoxication were collected from donor groups to carry out the fecal transplant. Our objective was to transplant IMB from animals showing withdrawal signs (as can be seen in Fig. 2B, before the first administration on the sixth day, the animals begin to present significant differences in these withdrawal signs). We homogenized 0.2 g fecal samples in sterile tubes with 1 ml buffer (50% glycerol, 50% saline solution and 0.1% L-cysteine) [25] and were stored at −80 °C until use [26]. A pool was prepared with the fecal samples of the intoxicated donor animals and another with the samples of the control animals. Recipient groups received a 0.3 ml microbiota transplant daily for six days [27]. Fecal transplants began the next day at the end of ABX treatment and were administered to three groups: alcohol microbiota recipients (*n* = 22), control microbiota recipients (*n* = 22) and buffer recipients (no microbiota, *n* = 22). Half of the rats in each group had previously been treated with ABX.

### Voluntary alcohol consumption

To study voluntary alcohol consumption in recipient animals, we used the “Drinking In the Dark-Multiple Scheduled Access” paradigm [28, 29]. The first period of alcohol consumption (10 days) began on the second day of fecal transplant. Rats were presented with two identical bottles in their home cages, one containing alcohol (10%) and another containing water. This paradigm involved three 1-h access sessions during the dark cycle, with the first session initiated at lights out and each subsequent period of access separated by 2-h of alcohol deprivation.

### Motor activity

To investigate whether the microbiota transplant had changed the spontaneous activity of the animals we assessed the motor activity of the recipient animals seven days after the first period of alcohol consumption. Locomotor activity was assessed for 30 minutes using six custom-made 40 × 35 × 35 cm rectangular boxes equipped with eight photocells [30].

### Collection of rat fecal samples for the bacterial analysis

Rat fecal samples for the bacterial analysis were taken at the following time points: the feces of the donor groups just after the last alcohol intoxication or the control treatment (t0). The feces of the recipient groups were taken after the last antibiotic or their respective vehicle treatment (t1) and before exposure to alcohol. A second sample was taken on the last day of alcohol consumption, just prior to its sacrifice (t2).

### DNA extraction and processing

DNA extraction from human and rat fecal samples (180–200 mg) was performed using the QIAamp^®^ DNA Stool Mini Kit (Qiagen France S.A.S.) following the manufacturer’s instructions. DNA concentration and purity were determined by absorbance at 260 nm (A260) and the A260/A280 ratio, respectively, using a NanoDrop spectrophotometer (NanoDrop TM One Spectrophotometer, Thermo Fisher Scientific Inc., Spain). DNA samples were sent to StarSEQ^®^ GmbH (Mainz, Germany) for the analysis of The V4–V5 hypervariable regions of the bacterial 16 S rRNA gene, amplified from the isolated DNA using the primer combination 515F–909 R. The Illumina MiSeq System was used to sequence DNA products of this PCR amplification and the 16 S metagenomics analysis was performed by the Illumina App.

### Statistical analysis

The analysis of the fecal microbiome was carried out in two steps. In the first step, operational taxonomic units (OTUs) were used for a more qualitative analysis approach of the samples (e.g., Evenness and Jaccard distance indices). These data were analyzed with the QIIME 2 platform. Alpha diversity was measured using Evenness (a measure of community evenness) and additionally the Shannon’s diversity index (a quantitative measure of community richness). Beta diversity was measured based on Jaccard distance (a qualitative measure of community dissimilarity) and the principal coordinates analysis (PCoA) plots were generated using Emperor for this Beta diversity metric. In the second step, for the quantitative analysis of the relative abundance of bacteria of each sample of the fecal microbiome it was used a proprietary algorithm from Illumina^®^ that provides a species-level classification for paired end reads. Benjamini–Hochberg false discovery rate (FDR) correction for multiple comparisons at each level was applied separately. FDR (*q*-value) <0.10 was considered significant. The taxonomy database is an Illumina-curated version of the Greengenes database (database/13_5). The statistical analysis was performed using the SPSS statistical software package (version 20.0) for Windows (Chicago, IL, USA). The behavioral data were analyzed using two-way mixed ANOVAs (within-subjects: consecutive days; between-groups: either antibiotic treatment and/or type of fecal microbiota transplant). Tukey’s post hoc analysis was used to compare more than two groups.

## Results

### Alcohol consumption, type of stool and bacteria in humans

**Fig. 1:**
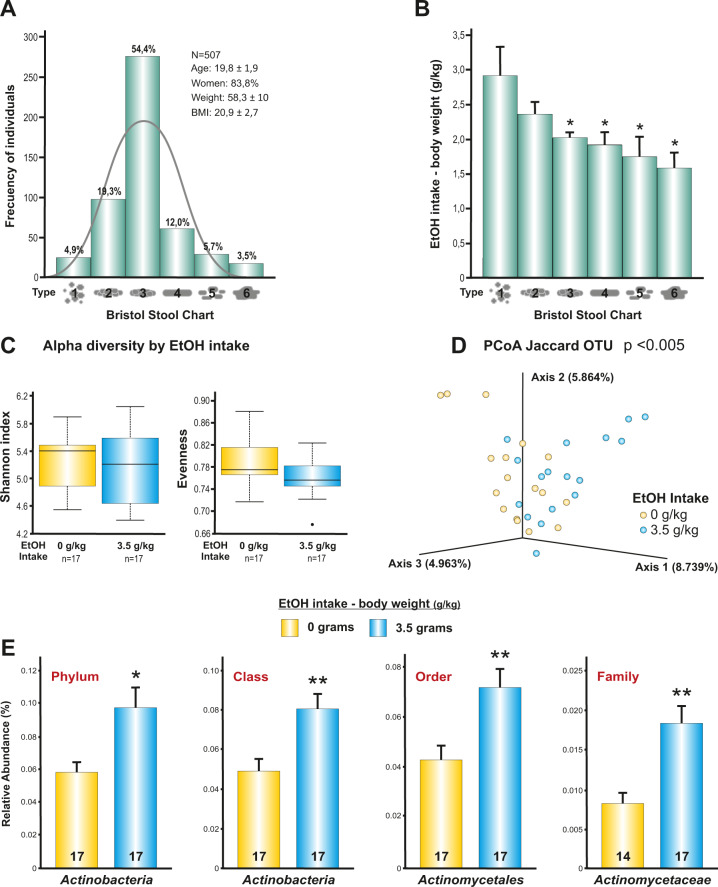
Association between stool type, alcohol consumption and IMB in human participants. **A** Distribution of the stool samples of university students according to the Bristol Stool Scale. **B** Association between heavy episodic drinking (g/kg BW) and stool type. *p < 0.05 compared to stool type number 1 (*n* = 507). **C** Alpha diversity did not differ significantly between groups. **D** Beta diversity showed significant differences between the groups (Jaccard distance index, *p* < 0.005). **E** Significant changes in the relative abundance of IMB. *Actinobacteria* was the main taxa affected by alcohol consumption. *p < 0.05; **p < 0.01 compared to non-alcohol group. **B**, **E** graphs represent mean ± s.e.m. Source data are provided as a Source Data file.

Stools were classified according to the Bristol Stool Scale. The distribution of the stool type in our sample of 507 university students was very close to the expected normal distribution. As shown in Fig. 1A, more than 50% reported having stool type number 3. Although the Bristol Stool Scale also considers a type number 7, which is completely liquid, in our sample there was not a single case. We examined the association between stool type and alcohol consumption (g/kg BW). We found that there was an association between stool type and the amount of alcohol consumed. Individuals who drank more alcohol tended to have a stool type number 1 (one-way ANOVA *F*_(5,506)_ = 3.06, *p* < 0.05). Furthermore, a linear relationship was observed between the type of stool and alcohol consumption. Those with the lower consumption were more likely associated with stool type number 6 (Fig. 1B).

Then, in order to investigate the effect of alcohol consumption on bacterial populations, we selected the only 17 subjects who reported that they did not drink any alcohol (pure controls for alcohol consumption) and the 17 subjects who consumed more alcohol (mean 3.5 g/kg). It is important to note that we analyze dietary fiber composition and water intake between the groups because these variables can influence the stool type and the IMB composition, but no significant differences were observed (data not shown). Alpha diversity was not significantly different between those two groups according to the Shannon (*p* < 0.74) and Evenness (*p* < 0.09) indices (Fig. 1C). That means that bacteria were evenly distributed within each group. However, Beta diversity, which it considers the between-group differences in diversity, showed significant differences (*p* < 0.005, based on the Jaccard distance index) (Fig. 1D). Regarding the relative abundance of bacteria, we found that *Actinobacteria* was the bacteria that systematically differed between groups (Fig. 1E), showing an increase through all the taxonomic levels (*p* < 0.05) in the alcohol group, except at the genus and species levels.

### Microbiota transplant from alcohol-dependent animals and effect of antibiotic treatment

**Fig. 2:**
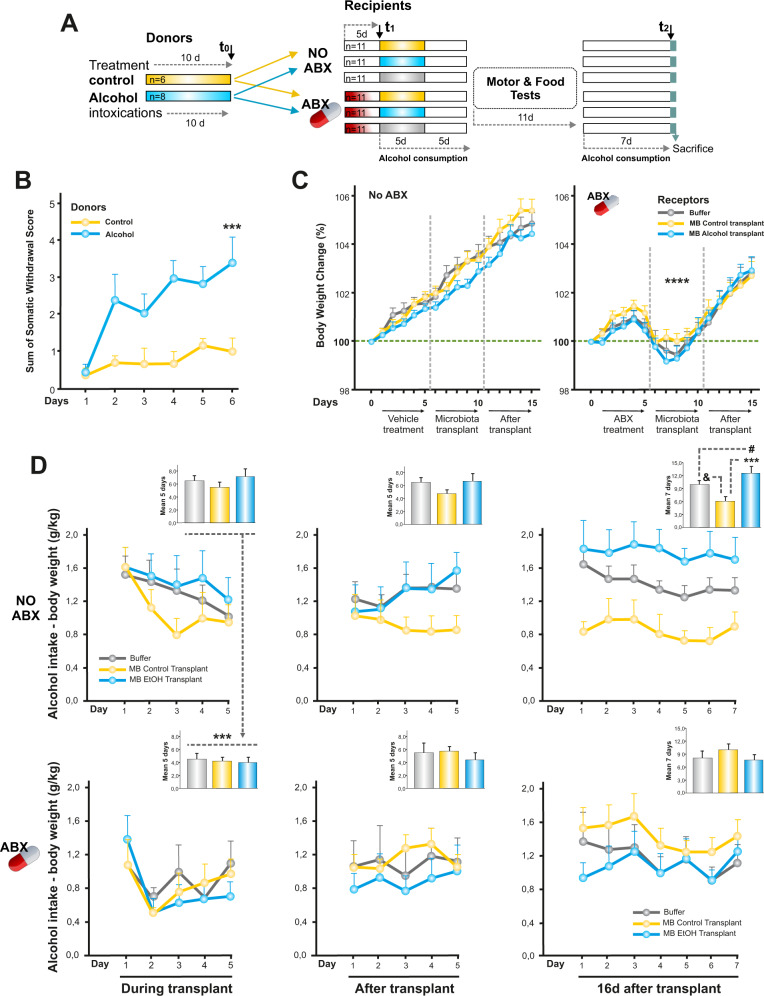
Effect of IMB transplant and antibiotics on alcohol consumption in rats. **A** Schematic representation of the experiments. Eight rats (blue) were intoxicated with alcohol for ten days. Another group was treated with the solution where the alcohol was dissolved (yellow, *n* = 6). The feces of these animals (donors of microbiota) were collected and transferred to other animals, half of which had previously been treated with an antibiotic cocktail (ABX, in red). Recipient groups (*n* = 11 per group) received each one a different microbiota transplant. A group of animals (in gray) was treated only with the buffer where the microbiota was dissolved. After 24 hours of the first transplant, animals were exposed to voluntary alcohol consumption for ten days. Next, the access to alcohol was withdrawn for 11 days. Finally, animals had access to alcohol for seven days and were finally sacrificed. **B** Donor alcohol-rats showed physical signs of withdrawal before the first alcohol intoxication of the sixth day, ***p < 0.005 compared to control group. **C** Sterilization of the rat intestine through a cocktail of antibiotics for five days resulted in weight loss, ****p < 0.001 compared to No ABX group. **D** Alcohol consumption evaluated during three periods of time. Inset panels represent the total amount of alcohol consumed in each period studied. ***p < 0.001, ^#^*p* < 0.05, ^&^*p* < 0.05. All the graphs represent mean ± s.e.m.

To investigate whether variations in IMB are a consequence or cause of increased alcohol consumption, we designed a series of experiments using rats (Fig. 2A). As expected, the induction of alcohol dependence via intragastric alcohol intubation leads to increasing signs of alcohol physical dependence evaluated by the sum of four observation scores (two-way ANOVA, between subjects, *F*_(1.12)_ = 18.22, *p* < 0.005) (Fig. 2B). The mortality rate was zero. On the other hand, antibiotic treatment caused a very significant reduction in the weight of the animals. This effect was independent of the microbiota type transplant received. Weight reduction began to be noticed on the fifth-last day of antibiotic treatment. The peak of weight loss, 3.1%, was shown on the second-third day after antibiotic treatment was finished (three-way ANOVA, *F*_(1.60)_ = 49.63, *p* < 0.001). Then, the animals began to gain weight normally (Fig. 2C).

Manipulations of the IMB lead to changes in voluntary alcohol consumption. The general three-way ANOVA showed a significant interaction between days, type of microbiota transplanted and antibiotic treatment (*F*_(32,960)_ = 1.59, *p* < 0.05). We considered each of the three periods in which alcohol consumption was evaluated individually. (1) During the five days in which the animals received the microbiota transplant, the antibiotic treatment reduced the consumption of alcohol by 34.3%, regardless of the type of transplant received (three-way ANOVA, *F*_(1.60)_ = 10.26, *p* < 0.005). (2) During the five days after the last microbiota transplant, no significant differences were observed between groups. (3) After 16 days from the last transplant, an interaction was observed between the type of transplant and the antibiotic treatment (three-way ANOVA, *F*_(2.60)_ = 5.98, *p* < 0.005). Animals which were not given antibiotic treatment and that received the microbiota transplant from alcohol-dependent rats showed a higher alcohol consumption (27.4%), compared to the buffer group. On the other hand, in the animals transplanted with microbiota from the control group, a reduction in alcohol consumption was observed by 39.1% (two-way ANOVA, between subjects, *F*_(2.30)_ = 7.45, *p* < 0.005) (Fig. 2D). We carried out additional analyses to guarantee the veracity of the results. Supplementary Fig. 1 is linked to Fig. 2D, right panels. It shows that during the three one-hour cycles of alcohol exposure, for seven days, the group of animals not treated with antibiotics and who received the microbiota transplants of alcohol-dependent animals, systematically showed a level of alcohol consumption above the other two groups (Supplementary Fig. 1).

### Identifying antibiotic- and alcohol-induced modifications of IMB

**Fig. 3:**
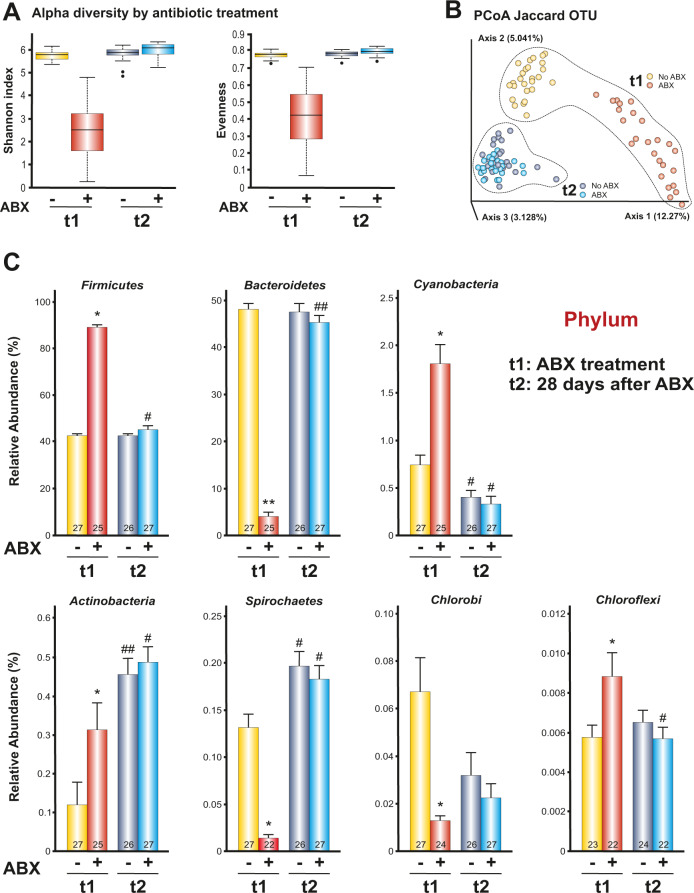
Effect of antibiotics and alcohol on IMB in rats. Antibiotics cause significant differences in Alpha diversity (*p* < 0.0001) (**A**) and Beta diversity (Jaccard distance index, *p* < 0.001) (**B**). This effect disappeared after four weeks. **C** Effects of the antibiotic treatment and alcohol consumption in relative abundance at phylum level. Significant differences between groups treated with antibiotic and non-antibiotic in t1: *p < 0.05; **p < 0.01. Significant differences within the same group between t1 and t2: ^#^*p* < 0.05; ^##^*p* < 0.01. **C** graph represents mean ± s.e.m. Source data are provided as a Source Data file.

We aim to identify the most significant changes induced by the antibiotic treatment and alcohol intake on the rat bacterial populations. For this purpose, we collected fecal samples at two times: “t1” just after antibiotic/vehicle treatment; and “t2” four weeks later in which animals could drink alcohol (see Fig. 2A). Alpha and Beta diversity were significantly different between “t1” and “t2” according to the Shannon (*p* < 0.0001), Evenness (*p* < 0.0001) and Jaccard distance (*p* < 0.001) indices (Fig. 3A, B). After four weeks, the effects of the antibiotic treatment disappeared. However, it is important to note that the IMB never returned to the levels of the animals free of antibiotic treatment in “t1” (NO-ABX group). Instead, the animals treated with antibiotics, four weeks later, managed to restore their gut microbiota to the same levels as the NO-ABX group. This would be the effect of alcohol on IMB (Fig. 3A, B and Supplementary video 1). Antibiotics significantly altered the bacteria at all taxonomic levels. To summarize, Fig. 3C shows only the effects at the level of phylum at t1 and t2. The use of antibiotics increased the relative abundance of *Firmicutes*, *Cyanobacteria, Actinobacteria* and *Chloroflexi* while dramatically reducing the abundance of *Bacteroidetes, Spirochaetes* and *Chlorobi*. *Firmicutes* and *Bacteroidetes* recovered their levels after four weeks of antibiotic treatment, but the same did not happen with *Cyanobacteria*, *Actinobacteria* and *Spirochaetes*. The latter phyla would be affected by the rat´s alcohol consumption.

### Microbiota transplant-induced reduction in locomotor activity

**Fig. 4:**
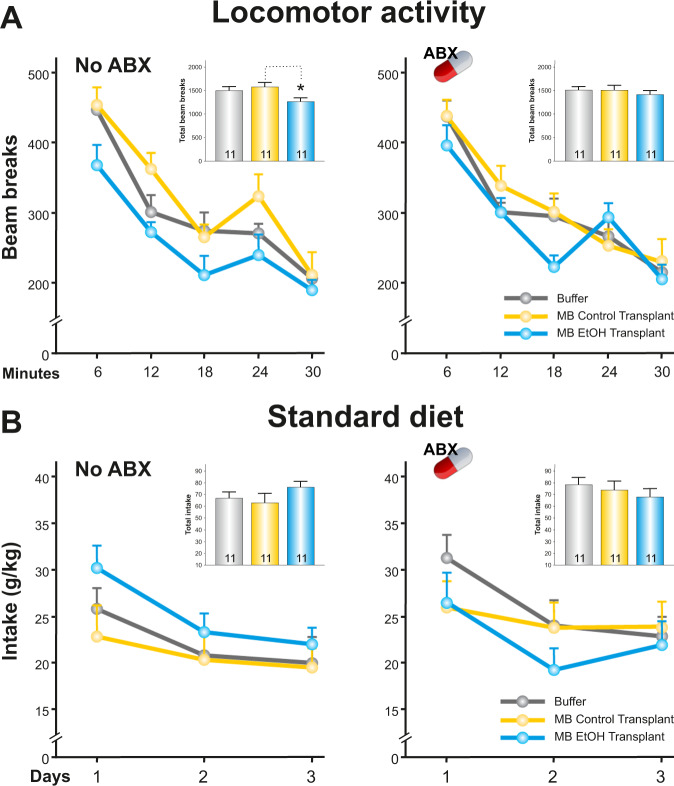
Evaluation of locomotor activity. Animals that received a microbiota transplant of animals treated with alcohol showed a reduction in their locomotor activity (**A**). This effect was not observed in the group of animals that was previously treated with antibiotics. There was not a significant difference in the intake of the standard diet (**B**). *p < 0.05 compared to MB Control Transplant group. All the graphs represent mean ± s.e.m.

We also study the spontaneous locomotor activity of rats and the intake of the standard diet. Unexpectedly, we found that the spontaneous activity of the animals not treated with ABX was reduced in the group of animals that received the fecal microbiota transplant of alcohol-dependent animals (total one-way ANOVA, *F*_(2.32)_ = 3.51, *p* < 0.05) (Fig. 4A). This did not occur in animals that were treated with antibiotics. Any group of animals did not differ significantly in the intake of the standard diet (Fig. 4B).

### Study of IMB in donor and recipient animals

**Fig. 5:**
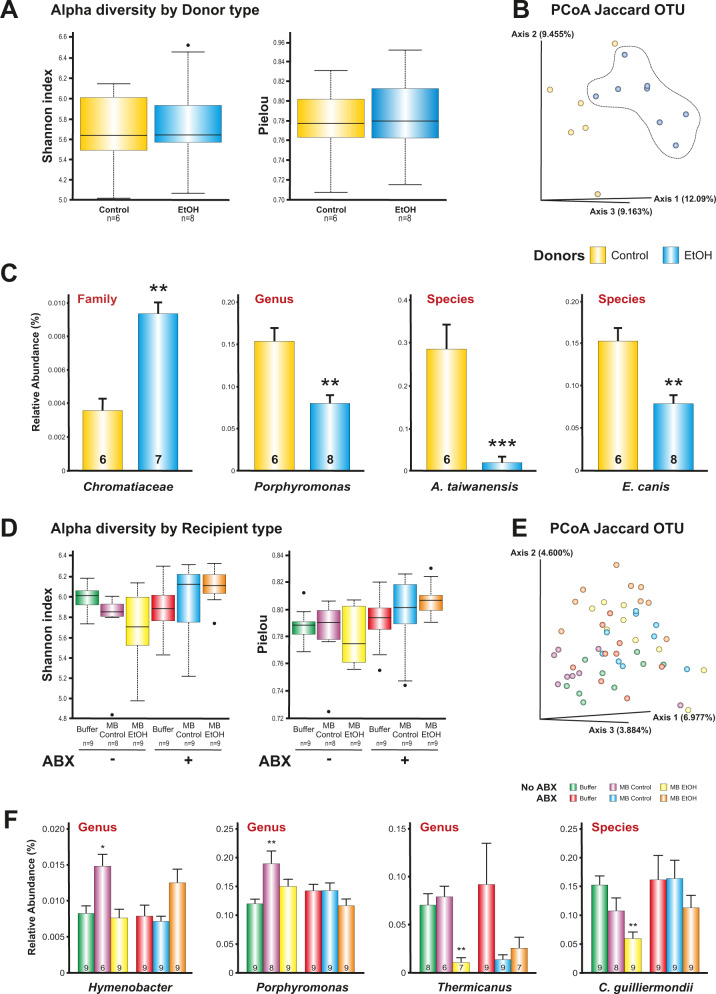
IMB composition in donor and recipient animals. Between alcohol- and control-donor groups there were not significant differences in Alpha diversity (**A**) but in Beta diversity (Jaccard distance index, *p* < 0.001) (**B**). Significant differences in relative abundance of bacteria between alcohol- and control-donor groups are shown in **C** (**p < 0.01; ***p < 0.001 compared to Control Group). Alpha diversity was not significant (*p* = 0.09) between recipient groups (**D**) but it was in Beta diversity (*p* < 0.001) (**E**). Relative abundance of bacteria in recipient groups are shown in **F**. *p < 0.05; **p < 0.01 compared to the respective Buffer groups, within each category (ABX, No ABX); **C**, **F** graph represent mean ± s.e.m. Source data are provided as a Source Data file.

There were no differences in Alpha diversity between alcohol- or control-donor groups (Fig. 5A). There were differences in Beta diversity showing alterations in the IMB induced by repeated alcohol intoxications (Fig. 5B*p* < 0.01). Alcohol led to a relative decrease in *A. taiwanensis* and *E. canis* species. At the Genus level, we found a relative decrease of *Porphyromonas*. Finally, at the Family level, we observed a relative increase of *Chromatiaceae* (Fig. 5C). In fecal transplant recipient groups, there were not observed statistical differences in Alpha diversity (*p* = 0.09, Fig. 5D) but significant differences in Beta diversity (*p* < 0.001, Fig. 5E). The pairwise PERMANOVA results are included as Supplementary Table 1. Due to the multiple interactions between the different groups, Fig. 5F only shows the differences compared to the respective Buffer groups, within each category (ABX, No ABX).

## Discussion

In this study, we went one step further on the role of the intestinal microbiota (IMB) in human physiology. We demonstrate that the motivation for alcohol consumption partially depends on the content of the IMB. In the first part of the study, we examined the phenotype of human feces through the Bristol Stool Scale, showing that heavy episodic drinking was associated with a specific type of stool. Those who consumed more alcohol had more prevalently stool type I, described as separate hard lumps, like nuts-hard to pass. This is an unexpected result since excessive alcohol consumption is usually associated with diarrhea [31]. The results obtained suggest that alcohol would lead to a reduction in bowel motility, causing a longer stay of food in the intestine and absorbing virtually all the water contained in those foods, leading to changes in stool consistency. This would be in accordance with the data obtained by Wegener et al., who showed an abnormally delayed gastric emptying in chronic alcoholics [32]. As long as 44–54% of the dry extract of the feces corresponds to populations of bacteria [33, 34], it would be plausible to assume that major alterations in IMB composition could partially be reflecting variation in stool consistency. Some researchers have verified this and stool consistency has been associated with gut microbiota richness, composition, enterotypes and bacterial growth rates [35]. These authors have hypothesized that transit time may act as a selective force on gut bacteria, favoring specific types of bacteria, based on their growth rate. Essentially, we found an increase in the abundance of *Actinobacteria* in the alcohol-group. This effect was found at all taxonomic levels except at the genus and species levels, where this effect was diluted. Therefore, the effect should be considered an effect on the “*Actinobacteria* ecosystem”, integrated at a higher taxonomic level, instead of the effect on individual species. Other authors have also found an increase of *Actinobacteria* in the mouse cecum after alcohol treatment [36–38]. Interestingly, according to Vindegaard et al., [39] the most consistent microbiota changes found in some psychiatric patients was a tendency towards higher abundance of *Actinobacteria*. It is important to note that analysis of our results in the human study indicates an association between alcohol consumption and bacterial abundance, which does not imply causality. On the other hand, alcohol consumption was calculated through a questionnaire asking the type of alcoholic beverage, the amount consumed, and the time spent in each episode of drinking. Although these data allowed us to estimate alcohol consumption, no standardized questionnaires were used for this purpose.

Given these results, we wonder if the IMB could influence alcohol consumption. To resolve this issue, we used an animal model of microbiota fecal transplant and the use of antibiotics. One of the first results obtained was that the use of a cocktail of antibiotics caused a significant reduction in alcohol consumption. Concerning this, numerous articles have explored a possible disulfiram-alcohol reaction produced by the administration of metronidazole. However, experimental articles and recent reviews [40–42] indicate that there is no evidence that this compound alters the alcohol metabolism pathway. On the other hand, similar data have been observed by Ezquer et al., who showed a reduction in voluntary alcohol intake after the administration of the other two antibiotics present in the cocktail used (neomycin and polymyxin B) [43].

In line with previous reports [44], the most remarkable result was that animals receiving a fecal transplant of alcohol-dependent animals, consumed more alcohol than animals that received a fecal transplant from control animals or those that were treated with the buffer where the feces were dissolved. However, this effect was not immediate. It was observed after two weeks of the last fecal transplant. This would have two possible explanations. The first, is that the incorporation of a new microbiota needs time to establish itself and lead to changes in behavior. The second, is that there is an interaction between the new microbiota received and alcohol consumption, producing a synergistic effect. We consider this second hypothesis more plausible. The new bacterial ecosystem would be a predisposing factor: when alcohol is added, this ecosystem would produce a feed-back system, somehow favoring the abundance of bacteria most benefited by alcohol consumption. More speculative would be to affirm that the interaction between the new bacterial ecosystem will modulate the host’s behavior to look for alcohol. It would be a sort of example of what it has been called, *the extended phenotype*, term coined by R. Dawkins [45] and promoted concept by R. Sapolsky [46] among other authors.

On the other hand, the observed reduction in spontaneous locomotor activity of rats that received the fecal microbiota transplant of alcohol-dependent animals, would be suggesting that the changes induced by this microbiota would affect the host in a more global way. Also, it could be possible that the IMB would be affecting the functioning of the brain reward system, since there is evidence that alterations in IMB can influence dopaminergic neurotransmission in the mesocorticolimbic circuit [47]. In addition, any substance that produces a reduction in motor activity is associated with a blockage of the dopaminergic reward system [48, 49].

Regarding the examination of intestinal bacteria, we observe that antibiotics caused, as expected, dramatic changes in the bacterial ecosystem. For example, compared to the control group, the phylum of *Bacteroidetes* reduced its relative abundance by 625%. However, these changes were almost completely reversed at four weeks after ABX treatment. *Firmicutes* and *Bacteroidetes*, which form more than 90% of the population of bacteria in rodents (and in humans) [50, 51] were restored. But other phyla, such as *Actinobacteria*, on the contrary, increased. It should be noted that the animals were exposed to alcohol once the antibiotic or vehicle treatment was finished. This suggests that the increase in the abundance of *Actinobacteria*, as seen with humans, could be a consequence of alcohol consumption. This link between alcohol exposure and elevation in phylum *Actinobacteria* has already been shown in earlier studies in mice [52]. To determine which bacteria were responsible for the increase in alcohol consumption in recipient animals, we analyzed the IMB of donor and recipient animals. It would be expected that if the distinct IMB composition were responsible for alcohol consumption, such modifications would be found in both groups. However, this was hard to find. According to our data, one of the most promising candidate would be the genus *Porphyromonas*. This genus is found to be significantly less abundant in alcohol donor animals and alcohol recipients (Fig. 5). As our data show relative abundance, the decrease of genus *Porphyromonas* would imply an increase of other bacteria. According to this, alcohol consumption would favor the growth of these bacteria. We have not found a specific genus that increases its relative abundance in alcohol-donors nor in the group of recipients with the highest alcohol consumption. However, the percentage of abundance lost by genus *Porphyromonas* could have been occupied by several bacteria, without showing a significant individual increase. Once the results of the two experiments have been described, we think that the main concern presented by the animal study is that it was carried out only in male rats. However, the human study involved subjects of both sexes. In fact, 83% of the samples came from women. Performing the studies under similar gender conditions would allow obtaining stronger associations between results.

In summary, our study represents a significant advance in the understanding of the role of IMB in motivated behavior. More specifically, our results on fecal transplantation show that IMB can help to explain differences in alcohol consumption among individuals and could be a potential target to manage alcohol abuse. This has already been previously suggested by other authors [53], however, the bacteria responsible for the observed effect, directly or indirectly, remain to be discovered.

## Supplementary information

Supplementary information

Gut microbiota and alcohol

## Data Availability

The datasets generated during the current study are available from the corresponding author on reasonable request. Figures that have associated raw data are: 1, 3, 5 and Supplementary Video 1.

## References

[1] Lloyd-Price J, Abu-Ali G, Huttenhower C (2016). The healthy human microbiome. Genome Med..

[2] Donaldson GP, Lee SM, Mazmanian SK (2016). Gut biogeography of the bacterial microbiota. Nat Rev Microbiol..

[3] O’Hara AM, Shanahan F (2006). The gut flora as a forgotten organ. EMBO Rep..

[4] Barrett E, Ross RP, O’Toole PW, Fitzgerald GF, Stanton C (2012). γ-Aminobutyric acid production by culturable bacteria from the human intestine. J Appl Microbiol..

[5] Franchi L, Kamada N, Nakamura Y, Burberry A, Kuffa P, Suzuki S (2012). NLRC4-driven production of IL-1β discriminates between pathogenic and commensal bacteria and promotes host intestinal defense. Nat Immunol..

[6] Neuman H, Debelius JW, Knight R, Koren O (2015). Microbial endocrinology: the interplay between the microbiota and the endocrine system. FEMS Microbiol Rev..

[7] Yano JM, Yu K, Donaldson GP, Shastri GG, Ann P, Ma L (2015). Indigenous bacteria from the gut microbiota regulate host serotonin biosynthesis. Cell..

[8] Uzbay T (2019). Germ-free animal experiments in the gut microbiota studies. Curr Opin Pharmacol..

[9] Rogers GB, Keating DJ, Young RL, Wong ML, Licinio J, Wesselingh S (2016). From gut dysbiosis to altered brain function and mental illness: mechanisms and pathways. Mol Psychiatry..

[10] Wang B, Yao M, Lv L, Ling Z, Li L (2017). The Human Microbiota in Health and Disease. Engineering..

[11] Substance Abuse and Mental Health Services Administration. Key substance use and mental health indicators in the United States: Results from the 2018 National Survey on Drug Use and Health (HHS Publication No. PEP19-5068, NSDUH Series H-54). 2019; Rockville, MD: Center for Behavioral Health Statistics and Quality, Substance Abuse and Mental Health Services Administration. https://www.samhsa.gov/data/

[12] Franzen M, Sadikaj G, Moskowitz DS, Ostafin BD, Aan Het Rot M (2018). Intra- and Interindividual Variability in the Behavioral, Affective, and Perceptual Effects of Alcohol Consumption in a Social Context. Alcohol Clin Exp Res..

[13] Norberg A, Jones AW, Hahn RG, Gabrielsson JL (2003). Role of variability in explaining ethanol pharmacokinetics: research and forensic applications. Clin Pharmacokinet..

[14] Whitfield JB, Zhu G, Duffy DL, Birley AJ, Madden PA, Heath AC (2001). Variation in alcohol pharmacokinetics as a risk factor for alcohol dependence. Alcohol Clin Exp Res..

[15] Collins SE (2016). Associations between Socioeconomic Factors and Alcohol Outcomes. Alcohol Res..

[16] Mayfield RD, Harris RA, Schuckit MA (2008). Genetic factors influencing alcohol dependence. Br J Pharmacol..

[17] Nixon K, McClain JA (2010). Adolescence as a critical window for developing an alcohol use disorder: current findings in neuroscience. Curr Opin Psychiatry..

[18] Leclercq S, Matamoros S, Cani PD, Neyrinck AM, Jamar F, Stärkel P (2014). Intestinal permeability, gut-bacterial dysbiosis, and behavioral markers of alcohol-dependence severity. Proc Natl Acad Sci USA..

[19] Leclercq S, De Saeger C, Delzenne N, de Timary P, Stärkel P (2014). Role of inflammatory pathways, blood mononuclear cells, and gut-derived bacterial products in alcohol dependence. Biol Psychiatry..

[20] Basis C, Moore N, Lolans K, Seekatz A, Weinstein R, Young V (2017). Comparison of stool versus rectal swab samples and storage conditions on bacterial community profiles. BMC Microbiol..

[21] Choo J, Leong L, Rogers G (2015). Sample storage conditions significantly influence faecal microbiome profiles. Sci Rep..

[22] Braconi S, Sidhpura N, Aujla H, Martin-Fardon R, Weiss F, Ciccocioppo R (2010). Revisiting intragastric ethanol intubation as a dependence induction method for studies of ethanol reward and motivation in rats. Alcohol Clin Exp Res..

[23] Macey DJ, Schultei G, Heinrichs SC, Koob GF (1996). Time-dependent quantifiable withdrawal from ethanol in the rat: effect of method of dependence induction. Alcohol..

[24] Davey P, Brown E, Charani E, Fenelon L, Gould I, Holmes A (2013). Interventions to improve antibiotic prescribing practices for hospital inpatients. Cochrane Database Syst Rev..

[25] Chu ND, Smith MB, Perrotta AR, Kassam Z, Alm EJ (2017). Profiling Living Bacteria Informs Preparation of Fecal Microbiota Transplantations. PLoS One..

[26] Tang G, Yin W, Liu W (2017). Is frozen fecal microbiota transplantation as effective as fresh fecal microbiota transplantation in patients with recurrent or refractory Clostridium difficile infection: A meta-analysis?. Diagn Microbiol Infect Dis..

[27] Kelly JR, Borre Y, O’ Brien C, Patterson E, El Aidy S, Deane J (2016). Transferring the blues: Depression-associated gut microbiota induces neurobehavioural changes in the rat. J Psychiatr Res..

[28] Bell RL, Rodd ZA, Smith RJ, Toalston JE, Franklin KM, McBride WJ (2011). Modeling binge-like ethanol drinking by peri-adolescent and adult P rats. Pharm Biochem Behav..

[29] Echeverry-Alzate V, Bühler KM, Calleja-Conde J, Huertas E, Maldonado R, Rodriguez de Fonseca F (2019). Adult-onset hypothyroidism increases ethanol consumption. Psychopharmacology..

[30] Roldán M, Echeverry-Alzate V, Bühler KM, Sánchez-Diez IJ, Calleja-Conde J, Olmos P (2018). Red Bull® energy drink increases consumption of higher concentrations of alcohol. Addict Biol..

[31] Haber PS, Kortt NC (2021). Alcohol use disorder and the gut. Addiction..

[32] Wegener M, Schaffstein J, Dilger U, Coenen C, Wedmann B, Schmidt G (1991). Gastrointestinal transit of solid-liquid meal in chronic alcoholics. Dig Dis Sci..

[33] Achour L, Nancery S, Moussata D, Graber I, Messing B, Flourié B (2007). Feacal bacterial mass and energetic losses in healthy humans and patients with a short bowel syndrome. Eur J Clin Nutr..

[34] Rose C, Parker A, Jefferson B, Cartmell F (2015). The Characterization of Feces and Urine: A Review of the Literature to Inform Advanced Treatment Technology. Crit Rev Environ Sci Technol..

[35] Vandeputte D, Falony G, Vieira-Silva S, Tito RY, Joossens M, Raes J (2016). Stool Consistency is strongly associated with gut microbiota richness and composition, enterotypes and bacterial growth rates. Gut..

[36] Lowe PP, Gyongyosi B, Satishchandran A, Iracheta-Vellve A, Ambade A, Kodys K (2017). Alcohol-related changes in the intestinal microbiome influence neutrophil infiltration, inflammation and steatosis in early alcoholic hepatitis in mice. PloS One..

[37] Moreira Júnior RE, de Carvalho LM, Pedersen A, Damasceno S, Maioli TU, de Faria A (2019). Interaction between high-fat diet and ethanol intake leads to changes on the fecal microbiome. J Nutr Biochem..

[38] Moreira Júnior RE, de Carvalho LM, Dos Reis DC, Cassali GD, Faria A, Maioli TU (2021). Diet-induced obesity leads to alterations in behavior and gut microbiota composition in mice. J Nutr Biochem..

[39] Vindegaard N, Speyer H, Nordentoft M, Rasmussen S, Benros ME (2020). Gut microbial changes of patients with psychotic and affective disorders: A systematic review. Schizophr Res..

[40] Tillonen J, Väkeväinen S, Salaspuro V, Zhang Y, Rautio M, Jousimies-Somer H (2000). Metronidazole increases intracolonic but not peripheral blood acetaldehyde in chronic ethanol-treated rats. Alcohol Clin Exp Res..

[41] Fjeld H, Raknes G (2014). Is combining metronidazole and alcohol really hazardous?. Tidsskr Nor Laegeforen..

[42] Steel BJ, Wharton C (2020). Metronidazole and alcohol. Br Dent J..

[43] Ezquer F, Quintanilla ME, Moya-Flores F, Morales P, Munita JM, Olivares B (2020). Innate gut microbiota predisposes to high alcohol consumption. Addict Biol..

[44] Zhao W, Hu Y, Li C, Li N, Zhu S, Tan X (2020). Transplantation of fecal microbiota from patients with alcoholism induces anxiety/depression behaviors and decreases brain mGluR1/PKC ε levels in mouse. Biofactors..

[45] Dawkins R. The extended phenotype, Vol. 8. Oxford: Oxford University Press; 1982.

[46] Dass SA, Vasudevan A, Dutta D, Soh LJ, Sapolsky RM, Vyas A (2011). Protozoan parasite Toxoplasma gondii manipulates mate choice in rats by enhancing attractiveness of males. PLoS One..

[47] González-Arancibia C, Urrutia-Piñones J, Illanes-González J, Martinez-Pinto J, Sotomayor-Zárate R, Julio-Pieper M (2019). Do your gut microbes affect your brain dopamine?. Psychopharmacology..

[48] Gaugler MN, Genc O, Bobela W, Mohanna S, Ardah MT, El-Agnaf OM (2012). Nigrostriatal overabundance of α-synuclein leads to decreased vesicle density and deficits in dopamine release that correlate with reduced motor activity. Acta Neuropatholgical..

[49] Parr-Brownlie LC, Hyland BI (2005). Bradykinesia induced by dopamine D2 receptor blockade is associated with reduced motor cortex activity in the rat. J Neurosci..

[50] Turnbaugh PJ, Ley RE, Mahowald MA, Magrini V, Mardis ER, Gordon JI (2006). An obesity-associated gut microbiome with increased capacity for energy harvest. Nature..

[51] Hegde S, Lin YM, Golovko G, Khanipov K, Cong Y, Savidge T (2018). Microbiota dysbiosis and its pathophysiological significance in bowel obstruction. Sci Rep..

[52] Xu Z, Wang C, Dong X, Hu T, Wang L, Zhao W (2019). Chronic alcohol exposure induced gut microbiota dysbiosis and its correlations with neuropsychic behaviors and brain BDNF/Gabra1 changes in mice. BioFactors..

[53] Leclercq S, Stärkel P, Delzenne NM, de Timary P (2019). The gut microbiota: A new target in the management of alcohol dependence?. Alcohol..

