# Supplementary material for: Gut microbiota and voluntary alcohol consumption
Source: Transl Psychiatry. 2022 Apr 7;12:146. doi: 10.1038/s41398-022-01920-2 (PMC8990010; doi:10.1038/s41398-022-01920-2)
Supplement: Supplementary file 1 — Supplementary information [file 41398_2022_1920_MOESM1_ESM.docx]

**Gut Microbiota and Voluntary Alcohol Consumption**

***Supplementary Information***

**Supplementary Figures**

**
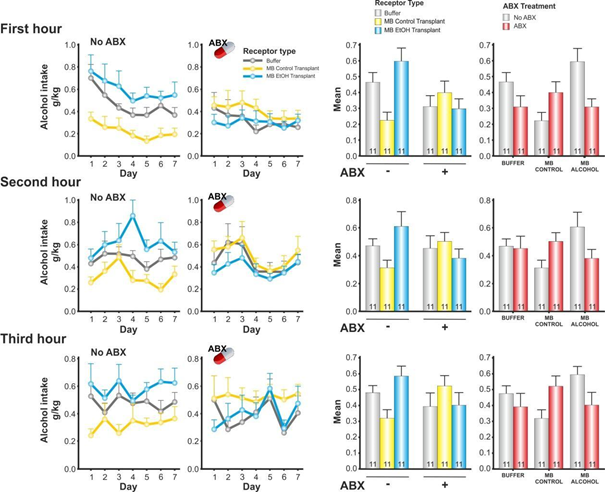
**

**Supplementary Figure 1** Alcohol consumption (in g/kg) during the first, second and third hour in which the animals had access to alcohol on the same day. Each access was separated by a two hour period free of alcohol. The panels on the left show the consumption broken down by days while those on the right show the average consumption of the seven days.

**Supplementary Tables**

| **Group 1** | **Group 2** | **Sample Size** | **Permutations** | **Pseudo-F** | **p-value** | **q-value** |
| --- | --- | --- | --- | --- | --- | --- |
| **ABX_Buffer** | ABX_Control | 18 | 999 | 1.122881 | 0.133 | 0.133000 |
|  | ABX_EtOH | 18 | 999 | 1.475463 | 0.002 | 0.005000 |
|  | No-ABX_Buffer | 18 | 999 | 1.132178 | 0.052 | 0.055714 |
|  | No-ABX_Control | 17 | 999 | 1.202998 | 0.048 | 0.055385 |
|  | No-ABX_EtOH | 18 | 999 | 1.356574 | 0.012 | 0.018000 |
| **ABX_Control** | ABX_EtOH | 18 | 999 | 1.336969 | 0.004 | 0.008571 |
|  | No-ABX_Buffer | 18 | 999 | 1.379113 | 0.002 | 0.005000 |
|  | No-ABX_Control | 17 | 999 | 1.275600 | 0.017 | 0.023182 |
|  | No-ABX_EtOH | 18 | 999 | 1.317666 | 0.024 | 0.030000 |
| **ABX_EtOH** | No-ABX_Buffer | 18 | 999 | 1.935745 | 0.001 | 0.003750 |
|  | No-ABX_Control | 17 | 999 | 1.817232 | 0.001 | 0.003750 |
|  | No-ABX_EtOH | 18 | 999 | 1.423123 | 0.011 | 0.018000 |
| **No-ABX_Buffer** | No-ABX_Control | 17 | 999 | 1.322543 | 0.001 | 0.003750 |
|  | No-ABX_EtOH | 18 | 999 | 1.833108 | 0.001 | 0.003750 |
| **No-ABX_Control** | No-ABX_EtOH | 17 | 999 | 1.544690 | 0.012 | 0.018000 |

**Supplementary Table 1.** Pairwise PERMANOVA results from beta diversity analysis from Figure 5.e.
